# Protection against neurodegeneration with low-dose methylene blue and near-infrared light

**DOI:** 10.3389/fncel.2015.00179

**Published:** 2015-05-12

**Authors:** F. Gonzalez-Lima, Allison Auchter

**Affiliations:** Department of Psychology and Institute for Neuroscience, University of Texas at AustinAustin, TX, USA

**Keywords:** mitochondrial respiration, near-infrared light, methylene blue, neuroprotection, neurotherapeutic potential

Neurons are metabolically protected against degeneration using low-level methylene blue and near-infrared light interventions. Both of these novel interventions act by a cellular mechanism involving enhancement of the electron transport chain in mitochondria, which promotes energy metabolism and neuronal survival (Gonzalez-Lima et al., 2014). Methylene blue preferentially enters neuronal mitochondria after systemic administration, and at low-doses forms an electron cycling redox complex that donates electrons to the mitochondrial electron transport chain. Low-level near-infrared light applied transcranially delivers photons to cortical neurons that are accepted by cytochrome oxidase, which causes increased cell respiration and cerebral blood flow. Breakthrough *in vivo* studies with these interventions suggest that targeting mitochondrial respiration may be beneficial for protection against different types of neurodegenerative disorders.

The purpose of this paper is to provide an update on the cellular mechanisms mediating the neuroprotective effects of low doses of methylene blue and near-infrared light, and to argue that the neurotherapeutic benefits of these two different interventions share the same cellular mechanism of action based on stimulation of mitochondrial respiration. Presented first is the explanation of the biochemical redox action of low-dose methylene as an electron cycler on the mitochondrial electron transport. Presented second is the explanation of the biophysical action of near-infrared light as a photon donor to cytochrome oxidase that also serves to stimulate mitochondrial electron transport. We finish with a comparison of these two interventions and how they share a common cellular mechanism with similar properties such as energy transfer, low-dose hormetic dose-responses, and enhanced capacity for oxidative metabolic energy production, which serve to protect nervous tissue from degeneration.

## Methylene blue as electron donor

**Figure 1:**
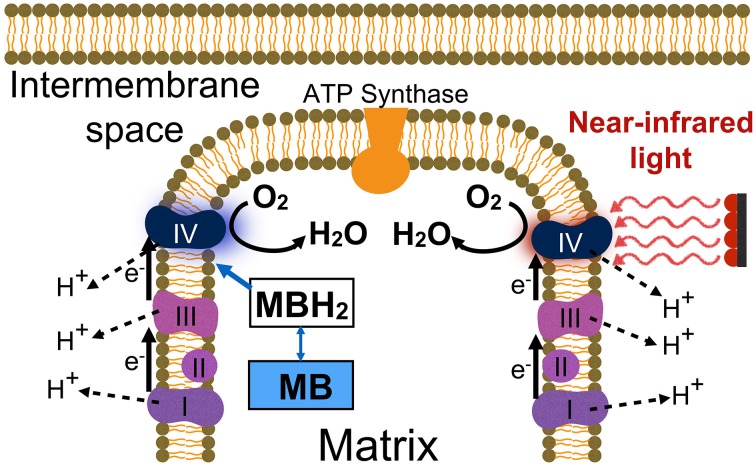
**Two neuroprotective interventions for enhancing mitochondrial respiration**. Low-dose methylene blue (MB) acts as an exogenous electron (e-) cycler, boosting oxygen consumption and cell respiration (molecular O_2_ reduced to H_2_O). Low-level red-to-near-infrared light directly energizes cytochrome oxidase (Complex IV) via photon absorption, facilitating its catalytic activity and leading to up-regulation of cytochrome oxidase levels. These interventions result in long-term increases in the amount of cytochrome oxidase in the electron transport chain by a process of enzymatic induction, which promotes oxidative energy metabolism and neuronal survival. Abbreviations: I–IV, refer to the four electron transport enzymatic complexes in the inner membrane of mitochondria; MB, is oxidized methylene blue (blue color); MBH_2_, is reduced methylene blue (colorless); H^+^, stands for the protons pumped by Complexes I, III, and IV that enter the mitochondrial matrix via ATP synthase, which results in ATP production.

Low-dose methylene blue stimulates mitochondrial respiration by donating electrons to the electron transport chain. This is possible by a unique auto oxidizing redox chemical property. Methylene blue is unique among chemicals for several important reasons. Foremost is the auto oxidizing property that allows methylene blue at low concentrations to form a redox equilibrium by cycling electrons (i.e., serving as both an electron donor and acceptor). This property permits the cycling of electrons from chemicals inside the mitochondrial matrix to electron transport proteins in mitochondria. These transport proteins act as acceptors for electrons donated by methylene blue in mitochondria. The final acceptor of electrons in the respiratory chain is oxygen, which is obtained from oxyhemoglobin transported in the circulation. Molecular oxygen becomes reduced to water in a reaction catalyzed by the mitochondrial enzyme cytochrome oxidase (Complex IV, cytochrome c oxidase). The electron transport chain is coupled with the biochemical process of oxidative phosphorylation, which leads to increased oxygen consumption and the formation of ATP from ADP (Figure 1). Under normal physiological conditions the electrons that enter the electron transport chain come from electron donor molecules such as NADH and FADH_2_. These molecules derive from the Krebs cycle conversion of the food we eat. Methylene blue at low concentrations serves as another source of electrons for the electron transport chain that is part of mitochondrial respiration, leading to increased cytochrome oxidase activity and oxygen consumption (Riha et al., 2005; Wen et al., 2011; Rojas et al., 2012a; Rodriguez et al., 2014).

In addition, recent functional magnetic resonance imaging (fMRI) studies show that systemically administered low-dose methylene blue can directly reduce oxygen to water and support cerebral metabolic rate of oxygen consumption and metabolic energy production in normoxic and hypoxic conditions *in vivo* (Huang et al., 2013). Since rapid activation of oxygen consumption leads to a local transient hypoxia, cytochrome oxidase changes from reducing oxygen to catalyzing the formation of nitric oxide (Poyton and Ball, 2011). This in turns leads to a hemodymamic vasodilation response that increases cerebral blood flow and brain glucose uptake *in vivo* (Lin et al., 2012). This metabolic cascade promotes bigenomic regulation of mitochondrial and nuclear genes whose expression up-regulate cytochrome oxidase protein subunits and holoenzyme levels in nervous tissue (Rojas et al., 2012a).

## Near-infrared light as photon donor

Near-infrared light from low-power lasers and light-emitting diodes (LEDs) stimulates mitochondrial respiration by donating photons that are absorbed by cytochrome oxidase, a bioenergetics process called photoneuromodulation in nervous tissue (Rojas and Gonzalez-Lima, 2013). Photons are packets of luminous energy from electromagnetic waves. The amount of energy delivered by a photon depends on the light wave frequency. Photons in the red-to-near-infrared frequency range of approximately 620–1150 nm penetrate to the brain and intersect with the absorption spectrum of cytochrome oxidase (Rojas and Gonzalez-Lima, 2011). The absorption of luminous energy by the enzyme results in increased brain cytochrome oxidase enzymatic activity and oxygen consumption (Rojas et al., 2012b). Since the enzymatic reaction catalyzed by cytochrome oxidase is the reduction of oxygen to water, acceleration of cytochrome oxidase catalytic activity directly causes an increase in cellular oxygen consumption. Because increased oxygen consumption by nerve cells is coupled to oxidative phosphorylation, ATP production increases as a consequence of the metabolic action of near-infrared light. This type of luminous energy can enter brain mitochondria transcranially, and—independently of the electrons derived from food substrates—it can directly photostimulate cytochrome oxidase activity.

Red-to-near-infrared light not only stimulates mitochondrial respiration and brain oxygen consumption when light is on. Light also has a longer lasting effect on metabolic capacity because its acceleration of cytochrome oxidase activity causes *enzymatic induction*, a process dependent on gene expression and protein synthesis that up-regulates the levels of cytochrome oxidase (Hayworth et al., 2010). For example, 1 day after a single session of photoneuromodulation, there are significantly higher levels of cytochrome oxidase enzyme in the rat brain (Rojas et al., 2012b). Indeed, behavioral effects in humans can still be observed at 2 and 4 weeks after a single transcranial near-infrared light treatment (Barrett and Gonzalez-Lima, 2013). This enzymatic induction provides a long-tern mechanism for increasing the oxidative metabolic capacity of neurons, which is manifested *in vivo* by increases in cerebral rates of oxygen consumption and blood flow to the brain (Uozumi et al., 2010; Rojas et al., 2012b).

## Cellular mechanisms of neuroprotection

While low-dose methylene blue and low-level near-infrared light may produce different pleiotropic cellular effects, both interventions cause a similar up-regulation of mitochondrial respiration with similar benefits to protect nerve cells against degeneration. First, both interventions increase the expression of brain cytochrome oxidase *in vivo* (Gonzalez-Lima et al., 2014). Methylene blue accomplishes this by supporting the electron transport chain, while near-infrared light does it by directly energizing cytochrome oxidase via photon absorption (Figure 1). Still, their primary cellular mechanism of action is the same: enhancement of mitochondrial respiration.

Second, both interventions share high bioavailability. Methylene blue readily crosses the blood-brain barrier, builds up inside neurons, and resides inside respiring mitochondria. Indeed, methylene blue injected to live animals was first used as a supravital stain of nervous tissue by Paul Ehrlich and Ramon y Cajal in the 1890s. In the modern case of near-infrared lasers used transcranially, the degree of penetration increases with longer wavelengths and pulse durations within the effective near-infrared spectrum range. For example, approximately 8–10% of luminous energy reaches the rat cerebral cortex and 1.7–2% reaches the human cerebral cortex (Gonzalez-Lima and Barrett, 2014).

Third, similar conditions affect their neural effects, such as the redox and activational status of the target tissue and the dose-response. Hormesis has been documented for the dose-responses of both methylene blue (Bruchey and Gonzalez-Lima, 2008) and near-infrared light (Huang et al., 2011). This phenomenon means that low doses produce opposite effects than high doses, and that intermediate doses may be ineffective. For example, while low doses of methylene blue are used to treat methemoglobinimia, high doses cause methemoglobinimia (Bruchey and Gonzalez-Lima, 2008). Hence it does not make sense to refer to methylene blue without specifying the dose level, as different doses produce opposite effects. For example, while high doses may inhibit tau aggregation and nitric oxide formation *in vitro*, they are toxic *in vivo* (Riha et al., 2005; O'Leary et al., 2010). But systemic low-doses (0.5–4 mg/kg) of methylene blue that stimulate mitochondrial respiration *in vivo* are safe and effective in both animals and humans (Rojas et al., 2012a). Similarly, only low-level near-infrared light is beneficial because higher doses become ineffective or produce opposite effects. The dosing for transcranial near-infrared light depends on multiple parameters besides wavelength, such as transmission, fluency, irradiance, number of fractions, pulsing, etc. (Huang et al., 2011). For example, forehead transcranial stimulation of the human cerebral cortex has been done effectively with a continuous wave 1064 nm laser at 60 J/cm^2^ (fluence), 250 mW/cm^2^ (irradiance) for 4 min, which corresponds to about 1.2 J/cm^2^ energy density reaching the cortical surface with a 2% transmission (Barrett and Gonzalez-Lima, 2013).

An effective mechanism of stimulation of mitochondrial respiration protects against neurodegeneration by increasing the oxidative metabolic energy capacity of neurons and reducing oxidative damage (Wen et al., 2011). With increases in the capacity to produce ATP by up-regulation of cytochrome oxidase, multiple secondary benefits accrue such as enhancement of neuronal metabolic energy and bigenomic responses, antiapoptotic signaling, DNA repair, mitogenic signaling, axonal sprouting, synaptogenesis and brain-derived neurotrophic factor (Martijn and Wiklund, 2010; Gomes et al., 2012; Poteet et al., 2012; Rojas and Gonzalez-Lima, 2013; Xuan et al., 2014). Low-doses of methylene blue and near-infrared light that up-regulate mitochondrial respiration *in vivo* have similar neuroprotective effects in multiple model systems featuring neurodegeneration. These include models of neurotoxicity (Zhang et al., 2006; Rojas et al., 2008, 2009a), ischemia (Yip et al., 2011; Watts et al., 2013; Auchter et al., 2014; Rodriguez et al., 2014), neurotrauma (Bittner et al., 2012; Oron et al., 2012; Quirk et al., 2012; Shen et al., 2013; Xuan et al., 2013; Zhang et al., 2014), neurocognitive and emotional impairment (Callaway et al., 2004; Gonzalez-Lima and Bruchey, 2004; Wrubel et al., 2007a,b; Riha et al., 2011), Alzheimer's disease (Callaway et al., 2002; Auchter et al., 2014), and Parkinson's disease (Rojas et al., 2009b; Wen et al., 2011).

Although most of the mechanistic studies have been undertaken in animal models, the *in vivo* neuroprotective benefits of low-dose methylene blue and near-infrared light have been documented in humans as well (Rojas et al., 2012a; Rojas and Gonzalez-Lima, 2013). For example, low-dose methylene blue in humans leads to neuroprotection and reversal of ifosfamide-induced encephalopathy (Turner et al., 2003), and it also improves treatment of bipolar and unipolar depressive disorders (Naylor et al., 1986, 1987). In addition, a recent controlled, randomized, double-blind study in phobic patients provided the first peer-reviewed report of improvement in human memory functions by low-dose methylene blue, specifically fear extinction memory and contextual memory (Telch et al., 2014). These human studies suggest that low-dose methylene blue may have potential therapeutic applications in neurology as a neuroprotective agent, and in psychiatry and clinical psychology to facilitate psychotherapeutic interventions. Similarly, low-level near-infrared light improved human neurological outcome after ischemic stroke (Lampl et al., 2007), and enhanced emotional and neurocognitive functions such as sustained attention and working memory in humans (Gonzalez-Lima and Barrett, 2014).

## Conclusion

We have described two very different chemical-physical interventions resulting in a similar cellular mechanism targeting mitochondrial respiration (Gonzalez-Lima et al., 2014). One entails fostering electron cycling by an auto-oxidizing redox chemical, and the other using photon absorption from near-infrared light. New *in vivo* evidence from animal models and human studies suggest that low-dose methylene blue and low-level near-infrared light share a common mechanism of enhancement of mitochondrial respiration that protects against neuronal degeneration in a broad range of animal models and human neurobehavioral disorders. We hope that this fascinating neuroprotective approach targeting mitochondrial respiration will stimulate more human studies of potential therapeutic applications in neurology and psychiatry.

### Conflict of interest statement

The authors declare that the research was conducted in the absence of any commercial or financial relationships that could be construed as a potential conflict of interest.
